# A Pilin Region Affecting Host Range of the *Pseudomonas aeruginosa* RNA Phage, PP7

**DOI:** 10.3389/fmicb.2018.00247

**Published:** 2018-02-16

**Authors:** Eun Sook Kim, Hee-Won Bae, You-Hee Cho

**Affiliations:** Department of Pharmacy, College of Pharmacy and Institute of Pharmaceutical Sciences, CHA University, Gyeonggi-do, South Korea

**Keywords:** *Pseudomonas aeruginosa*, RNA phage, PP7, type IV pilus (TFP), pilin, twitching

## Abstract

The host range of a phage is determined primarily by phage-receptor interaction. Here, we profiled the host range of an RNA leviphage, PP7 that requires functional type IV pilus (TFP) in order to enter into its host bacterium, *Pseudomonas aeruginosa*. Out of 25 twitching-proficient *P. aeruginosa* strains, 4 with group I pilin and 7 with group III pilin displayed PP7-resistance. The remaining 14 possessed group II pilin, which included 10 PP7-sensitive and 4 PP7-resistant strains, suggesting that only the strains with TFP consisted of a subset of group II (hence, group IIa) pilin were susceptible to PP7. The co-expression of the PAO1 (group IIa) pilin rendered all the strains susceptible to PP7, with the exception of the 4 strains with group I pilin. Moreover, the expression of PA14 (group III) and PAK (group IIb) pilin in the PAO1 *pilA* mutant restored the twitching motility but not the PP7-suceptibility. Site-directed and random mutation analyses of PAO1 pilin enabled us to identify a pilin mutant (G96S) that is fully functional but resistant to PP7 infection. This is due to the lack of any phage-receptor interactions, suggesting the structural properties of the β1-β2 loop in the variable region 2 of the group II pilin might be involved in PP7 infection.

## Introduction

The host range of a bacteriophage (phage) is the collection of bacterial hosts which allow a phage species to complete its life cycle using the host materials. This is determined by the balance between the bacterial resistance maneuvers and the counter-resistance adaptations of a phage. Bacterial resistance is generally exerted throughout the five steps of the phage life cycle including; phage adsorption, genome penetration, nucleic acid and protein synthesis, virion assembly, and phage release. One such example is adsorption resistance, which includes loss of phage receptors from hosts and the acquisition of physical barriers hiding receptors. This ultimately leads to a reduced interaction between the phage and the bacterium (Hammad, 1998; Høyland-Kroghsbo et al., 2013; Seed et al., 2014). Another example can be seen during host restriction occurring after phage adsorption. This includes blockage in genome penetration, superinfection immunity, restriction modification, and CRISPR (Lu and Henning, 1994; Hofer et al., 1995; McGrath et al., 2002; O’Driscoll et al., 2006; Barrangou et al., 2007; Hoskisson and Smith, 2007; Dupuis et al., 2013). These complex host strategies to resist phages may explain the strain-specificity of phage infection even in the same bacterial species and thus be one of the major obstacles significant to phage exploitation as antibacterials (i.e., phage therapy) or to protection from phage contamination in fermentation industry.

Small RNA phages belonging to the *Leviviridae* family infect several genera of Gram-negative bacteria. They have positive single-stranded (ss) RNA genomes within an icosahedral capsid (Persson et al., 2008). Their genomes, which are about 4,000 nucleotides in length, are typically composed of four genes, encoding maturation and coat proteins, a protein with lysis function, and replicase (van Duin and Tsareva, 2005). To date, the isolated ssRNA phages require various pili such as F plasmid-encoded pilus (for MS2 and Qβ) (Fiers et al., 1976; van Duin and Tsareva, 2005), other conjugative IncP plasmid-encoded pilus (for PRR1, C-1, Hgal1, and M) (Dhaese et al., 1977; Sirgel and Coetzee, 1983; Kannoly et al., 2012; Rumnieks and Tars, 2012), and chromosome-encoded type IV pilus (TFP) of *Pseudomonas aeruginosa* (for PP7) (Weppelman and Brinton, 1971). Based on the receptor characteristics, as well as their small RNA genome, particle sizes, and non-transducing characteristics, these pili-specific RNA phages need to be further investigated for their potential applicability to control Gram-negative ESKAPE pathogens including *Klebsiella pneumoniae*, *Acinetobacter baumannii*, *P. aeruginosa*, and *Enterobacter* species.

*P. aeruginosa* is an opportunistic human pathogen capable of epithelial infection in the airway and also becoming highly resistant to multiple antibiotics (Obritsch et al., 2004). These characteristics create an ideal model bacterium for phage therapy. More importantly, the TFP is involved not only in the interaction by various *P. aeruginosa* phages, but also in virulence and survival by directing adhesion and migration at the various surfaces in the host tissues or environmental habitats (Comolli et al., 1999; Heo et al., 2007; Burrows, 2012). As an initial step of the study regarding the TFP-associated alteration of the host spectrum in *P. aeruginosa* phages, we determined the host range of a TFP-specific leviphage, PP7 using the strains with group I, group II, or group III pilins (Kus et al., 2004; Harvey et al., 2009). A set of group II pilins were required for PP7-infection, the determinants of which are associated with the β1-β2 loop region that is flexibly protruding among the highly conserved structure of group II pilins.

## Materials and Methods

### Bacterial Strains and Culture Conditions

The bacterial strains used in this study were listed in **Table 1** and Supplementary Table S1. They were grown at 37°C using Luria-Bertani (LB) broth or on 2% Bacto-agar (Difco) LB plates. Antibiotics were used in the following concentrations (μg/ml): for *P. aeruginosa*, gentamicin (50), and carbenicillin (200); for *Escherichia coli*, gentamicin (10) and ampicillin (50).

**Table 1:** Host range and pilin grouping of twitching-proficient *Pseudomonas aeruginosa* strains.

Pilin group (number)	Strain^a^	PP7-sensitivity
Group I (4)	PMM23, PMM32, PMM41 PMM44	Resistant
Group IIa (10)	PAO1, PMM7, PMM11, PMM16, PMM18, PMM19, PMM21, PMM27, PMM49, 57RP	Sensitive
Group IIb (4)	PAK, PMM40, PMM50, PMM52	Resistant
Group III (7)	PA14, PMM1, PMM2, PMM3, PMM26, PMM39, PMM46	Resistant

**FIGURE 1:**
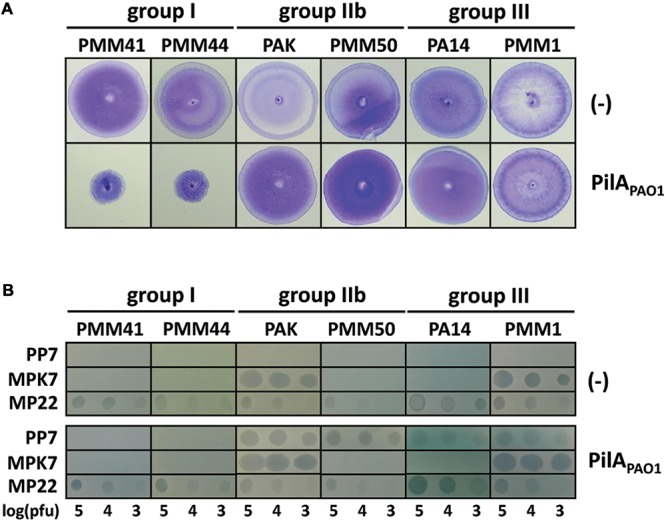
PAO1 pilin co-expression in PP7-resistant *Pseudomonas aeruginosa* strains. **(A)** Twitching motility of *P. aeruginosa* strains with either pUCP18 (–) or pUCP-pilA_PAO1_ (PilA_PAO1_) containing the PAO1 pilin coding region. Group I (PMM41 and PMM44), group IIb (PAK and PMM50) and group III (PA14 and PMM2) strains were used. Photographs were taken after 24 h using twitching assay plates. **(B)** PP7 lysates were spotted on the same strains as in A. Two other TFP-specific phages (MPK7 and MP22) were used as the controls (Bae and Cho, 2013). The numbers indicate the log(pfu) of the phage spots.

***^a^**underline denotes the strains used in **Figure 1**.*

### Preparation of Phage Lysates

Phage strains PP7, MPK7, and MP22 are enriched by the plate lysate method, using *P. aeruginosa* strain PAO1, as described elsewhere (Heo et al., 2007). Phage lysates were prepared from phage plaques on LB plates by adding phage buffer [10 mM MgSO_4_, 10 mM Tris (pH7.6), and 1 mM EDTA] and the phage particles were precipitated with 10% polyethylene glycol (average molecular weight, 8,000 Da) and 1 M NaCl at 4°C overnight. Then the precipitated phages were dissolved in phage buffer and titer of the phages was determined by serial dilution.

### Twitching Motility Assay

A single colony from overnight culture on LB agar plates was picked with a toothpick and stabbed to the bottom of a 3-mm thick 1.5% LB agar plate (Chung et al., 2014). After incubation at 30°C for 24 h, a twitching zone at the interface between the agar and the bottom was stained with 0.1% crystal violet.

### Phage Infection Assay

Phage infection was observed by the conventional spotting assay. Spotting of phage samples on the lawns of *P. aeruginosa* cells was performed as described previously (Heo et al., 2007), with some modifications. Droplets (3 μl) of serially diluted phage samples were spotted onto an LB medium top layer containing 0.7% agar and 50 μl of *P. aeruginosa* cells from the late logarithmic cultures (i.e., the optical density at 600 nm (OD_600_ of 1.0). The plates were incubated at 37°C for 16 h.

### Pilin Grouping of *P. aeruginosa* Strains

The *pilA* and adjacent sequences of *P. aeruginosa* were amplified by PCR. The *P. aeruginosa* strains were cultured on LB agar plates and 1.6 × 10^4^ cells were used as template of PCR reaction. The used primer sets were pilA-N1, pilA-C1, and pilA-C2 (Supplementary Table S2). The PCR products were subjected to sequencing and pilin grouping was performed based on the size of PCR products and the pilin sequence (Kus et al., 2004).

### Phylogenetic Analysis

Deduced amino acid sequences of PilA from *P. aeruginosa* strains with group II pilin were aligned in CLUSTAL_X (Thompson et al., 1997) using default parameters. Phylogenetic tree was generated with MEGA6 program with an output from a ClustalW alignment based on the neighbor-joining method (Kumar et al., 2001).

### Creation of the *pilA* Deletion Mutants

The *pilA*-deleted constructs for the pilin genes from PAO1, PAK, and PA14 strains were generated by 4-primer SOEing (splicing by overlap extension) PCR and the resulting PCR products were cloned into pEX18T (Heo et al., 2007). These plasmids were introduced into PAO1, PA14, and PAK strains by conjugation and then double-crossover deletion mutants were obtained by sucrose-resistance selection from the single-crossover cointegrates. The *pilA* deletion mutants were verified by PCR and twitching motility assay.

### Cloning of *pilA* and Complementation Experiments

The *pilA* genes were amplified from PAO1, PAK, and PA14 strains using the primers, pilA-N1 and pilA-C1 (Supplementary Table S2) and the resulting PCR amplicons were cloned into pUCP18 at the *Eco*RI and *Hin*dIII sites (Schweizer, 1991). The resulting plasmids were introduced into the *pilA* deletion mutants by electroporation (using a Bio-Rad MicroPulser^TM^) (Choi et al., 2006).

### Generation of Mutant Pilins

The random mutagenesis of the PAO1 pilin gene was performed by EMS (ethyl methanesulfonate) *in vitro*. Briefly, 500 μg of pUCP-pilA_PAO1_ DNA was incubated with 5% EMS and the ethylation was stopped by adding 5% sodium thiosulfate. The EMS-treated DNA was dialyzed and then introduced into the PAO1 *pilA* mutant by electroporation. Both the twitching motility and PP7 infectivity of transformants were evaluated to isolate a mutant pilin with two point mutations (A96T and G96S) out of∼6,000 tested transformant clones. Three point mutants (I90P, A95T, and G96S) at the β1-β2 loop region of the PAO1 pilin were generated by site-directed mutagenesis with 4-primer SOEing PCR. Each mutant construct was cloned into pUCP18 and then introduced into the PAO1 *pilA* mutant after sequence verification.

### Phage Adsorption Assay

Phage adsorption was performed as described (Lin and Schmidt, 1972). The PAO1 and its derivative bacteria were grown on tryptic soy (TS) agar plates at 37°C for 12 h and the cells (10^8^ cfu) of each bacterium were resuspended in 500 μl TS broth. Phage PP7 (3 × 10^7^ pfu) was added and allowed to adsorb to cells at 37°C for 5 min. The mixture was centrifuged at 500 × *g* to eliminate absorbed phages and the pfu of the supernatant was enumerated by plaque assay.

## Results

### Pilin Grouping and Determination of PP7-Susceptibility

We first determined the host-spectrum of PP7 using 25 twitching-proficient *P. aeruginosa* strains in our culture collection. We also evaluated their pilin groups based on the sequence variations in the DSL (disulfide-bonded loop) region (Kus et al., 2004; Harvey et al., 2009). As in **Table 1**, 14 strains contain group II pilin, 7 strains contain group III pilin, and 4 strains possess group I pilin. It is of marked interest that all the PP7-susceptible strains including PAO1 contained group II pilin (group IIa pilin, hereafter). Conversely, the other 15 strains were PP7-resistant, harboring group I pilin, group III pilin, or group II pilin (group IIb pilin, hereafter). This result suggested that PP7 may require a set of group II pilins for its infection through TFP.

### PAO1 Pilin Co-expression in PP7-Resistant *P. aeruginosa* Strains

To confirm that the host-spectrum of PP7 was associated with the group of the TFP pilin, a group IIa pilin in the strain PAO1 (PilA_PAO1_) was expressed in the *P. aeruginosa* strains. These bacteria are merozygotes likely expressing two alleles of pilins, and thus their twitching motilities needed to be measured to ensure the function of TFP upon the ectopic PilA_PAO1_ expression. The 21 strains whose endogenous pilin belongs to group II or III displayed comparable twitching motility and they were subsequently susceptible to PP7 infection (**Figure 1** and **Table 1**). In contrast, the 4 group I pilin-possessing strains were reduced in twitching and resistant to PP7 (**Figure 1**). It is interesting that MP22 is still infecting the group I-pilin strains expressing PilA_PAO1_, although it is unclear about the molecular mechanism of the twitching reduction of the strains. Nevertheless, it is quite clear that PilA_PAO1_ expression resulted in the sensitivity to PP7 in *P. aeruginosa* strains with group IIb or group III pilins, suggesting that the host spectrum of PP7 is more preponderantly associated with the pilin group than that of MPK7 is (**Figure 1B**).

**FIGURE 2:**
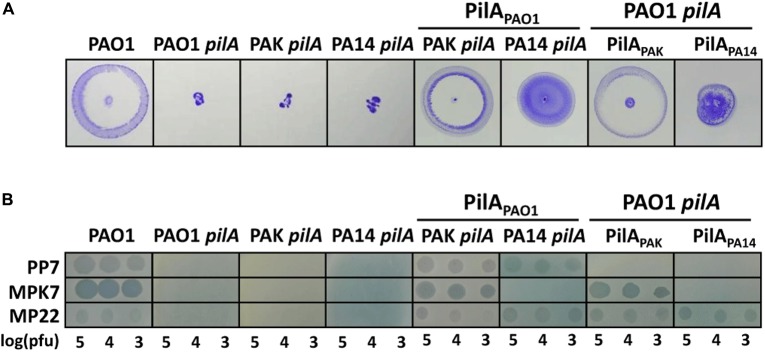
PAO1 pilin expression in *pilA* mutants of group IIb and group III *P. aeruginosa* strains. **(A)** Twitching motility of the *pilA* deletion mutants *(pilA)* of PAK (PAK *pilA*) and PA14 (PA14 *pilA*), which express the PAO1 pilin (PilA_PAO1_) and of the *pilA* mutant of PAO1 (PAO1 *pilA*), which express either the PAK pilin (PilA_PAK_) or the PA14 pilin (PilA_PA14_). Photographs were taken after 24 h using twitching assay plates. **(B)** PP7 lysates were spotted on the same strains as in A. TFP-specific phages, MPK7 and MP22, were used as the controls as in **Figure 1B**. The numbers indicate the log(pfu) of the phage spots.

This result was further verified by introducing PilA_PAO1_ into the *pilA* mutants of PAK (group IIb) and PA14 (group III). The in-frame deletion mutants for the pilin gene have been generated for PAK and PA14 (Heo et al., 2007; Bae and Cho, 2013; Chung et al., 2014). Upon introduction of PilA_PAO1_ into the pilin mutants, their phenotypes such as twitching motility and PP7-susceptibility were investigated (**Figure 2**). Their twitching defects were all rescued by the PAO1 pilin even in the PA14 mutant with group III pilin with its accessory gene (*tfpY*) intact (Kus et al., 2004; Chung et al., 2014). Based on these results, we have concluded that the PP7-susceptible pilins such as PilA_PAO1_ might be generally sufficient to drive PP7-susceptibility in *P. aeruginosa*, once the pilin is functionally incorporated into the TFPs.

### Heterologous Pilin Expression in PAO1 *pilA* Mutant

**FIGURE 3:**
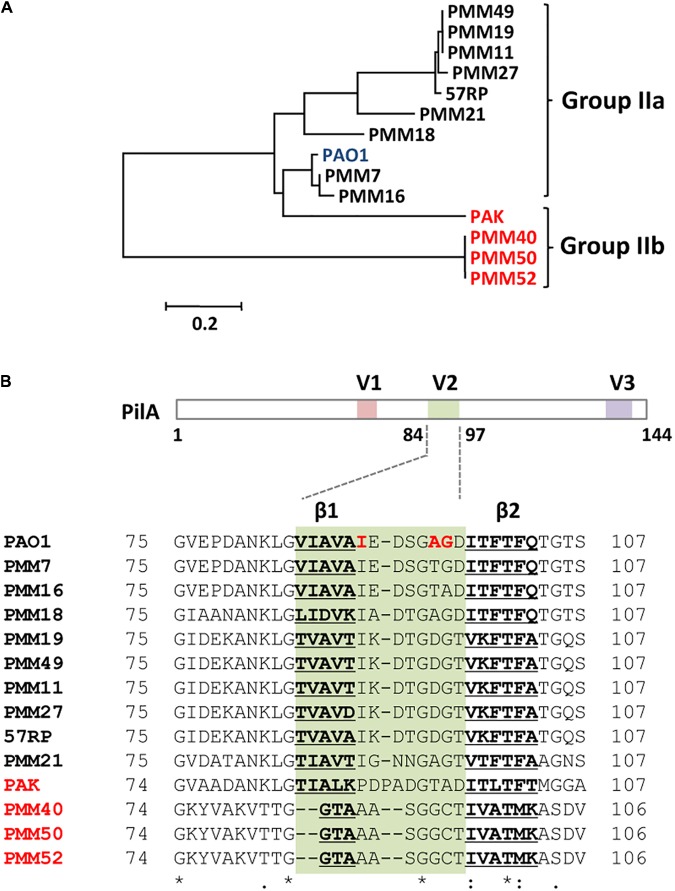
Sequence comparison of group II pilins from *P. aeruginosa* strains. **(A)** Phylogenetic tree of the group II pilins. Amino acid sequences of the 14 group II pilins in **Table 1** were aligned using ClustalW and the neighbor-joining tree has been generated by the MEGA5 program. Group IIb pilin strains are designated in red. **(B)** Schematic representation of the PAK pilin with three variable regions (V1, V2, and V3) and the partial sequences of the V2 region (green) from the *P. aeruginosa* strains in **A**, which encompass the two β-sheets (β1 and β2; bold and underlined) and the loop region between them (i.e., β1-β2 loop). The numbers indicate the amino acid position of each *P. aeruginosa* pilin. Identical residues in all the 14 pilins are designated by asterisk (^∗^) and the residues in the β1-β2 loop subjected to point mutation analyses are indicated in red for the PAO1 pilin (see **Figure 4**).

In order to confirm that the pilin variation is a primary determinant of PP7-susceptibility, we have introduced either the PAK (group IIb) or PA14 (group III) pilin into the PAO1 *pilA* mutant. Twitching motilities of PAO1 *pilA* mutant bacteria that possessed group IIb or III pilin appeared to be restored to the comparable level to that of the wild type (**Figure 2A**). In stark contrast, the *pilA* mutants expressing either PAK or PA14 pilin were PP7-resistant, although their sensitivities to other TFP-specific phages remained unchanged (**Figure 2B**). These results suggested that pilin variation may be the primary host barrier for PP7-susceptibility and that PAK pilin indeed belongs to the group IIb pilin despite the overall similarity with the group IIa pilins (**Figure 3**).

### Phylogenetic Relatedness of Group II Pilins of *P. aeruginosa* Strains

In an attempt to elucidate the pilin determinants in the PP7 susceptibility of the group II pilins, the amino acid sequences of group IIa and group IIb pilins were carefully compared by a multiple alignment from which the phylogenetic tree was generated (**Figure 3A**). Ten group IIa pilins (from the strains including PAO1) were clustered together, while the group IIb pilins (from the strains, PMM40, PMM50, and PMM52) except for the PAK pilin were closely clustered. The PAK pilin is noteworthy in that it is more closely clustered with the group IIa pilins than with the other group IIb pilins, although PAK strain is not susceptible to PP7 infection. This result of the group II pilin sequence comparison suggested that the difference between PAO1 and PAK pilins may make difference in regards to PP7 susceptibility. This would enable us to identify the actual determinants of PP7 susceptibility based on the sequence comparison of both group II pilins.

### Sequence Comparison of Group II Pilins

Based on the various regions of the PAK pilin as depicted in **Figure 3B**, three variable regions are suggested to be exposed out to the surface of the assembled TFPs (Hartung et al., 2011). Among the variable regions, the V2 region that is partially associated with the other two variable regions inside of the TFP surface appears the most protruding. This indicated that the V2 region might be likely involved in the interaction with the PP7 phage particles. Thus, the sequence compilation of the V2 region at the amino acid residues from 75 (or 74) to 107 (or 106) of the group II pilins was performed to identify the most variable residues within the V2 region (**Figure 3B**). The most variable region lies between the two β (β1 and β2) sheets, especially in the β1 sheet and the β1-β2 loop region. As a group IIa pilin, the PMM21 pilin shows some unexpected variations at this loop despite being closely clustered with the other group IIa pilins. To uncover the underlying mechanisms of the PP7-TFP interaction, the molecular details used to distinguish the sequence variations at the β1 sheet and the β1-β2 loop region (i.e., amino acid residues from 90 to 97 for PAO1 pilin) need to be further investigated.

### Identification of Mutant Pilins

**FIGURE 4:**
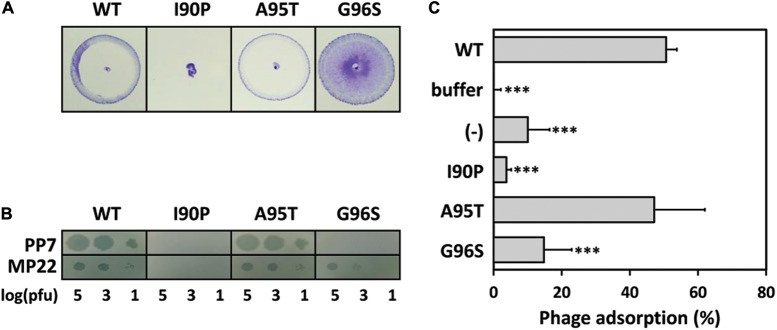
Expression of mutant pilins in PAO1 *pilA* mutant. **(A)** Twitching motility of the PAO1 *pilA* mutants expressing either the wild type pilin (WT) or one of the point mutants (I90P, A95T, and G96S) in the β1-β2 loop region as noted in **Figure 3B**. Photographs were taken after 24 h using twitching assay plates. **(B)** PP7 lysates were spotted on the same strains as in A. A TFP-specific phage, MP22 was used as the controls. The numbers indicate the log(pfu) of the phage spots. **(C)** Phage adsorption assay with PP7. Phages were incubated with the strains in A in parallel to the buffer control (buffer) and PAO1 *pilA* mutant (–). Unbound phages in the supernatant were measured by plaque assay to calculate the relative adsorption. Statistical significance for comparison with the WT result based on the Student’s *t*-test is indicated: ^∗∗∗^*P* < 0.001.

Due to the failure of domain swapping between PAO1 and PAK pilins to create the functional pilin derivatives (Supplementary Figure S1), we made a random chemical mutagenesis to find a PAO1 pilin mutant which remains functional in twitching but is lost in the PP7-TFP interaction. Ethyl methanesulfonate (EMS) mutagenesis that leads to G/C to A/T transition was exploited to screen for such PAO1 pilin mutants. Among the pilin mutants with PP7-resistance, one clone was obtained based on its ability to rescue twitching defect of the *pilA* mutant. This mutant was verified to contain two adjacent codon changes (GCG to ACG and GGT to AGT) at the β1-β2 loop region (i.e., A95T/G96S mutation), suggesting that the β1-β2 loop region involving these two residues might be critical in PP7-susceptibility. To further verify which mutation is critical for the mutant phenotype, we have generated two single mutants (A95T and G96S) of the PAO1 pilin. We also created another mutant (I90P) as a control, which turned out to be non-functional (**Figure 4A**). In contrast, the A95T and G96S mutants were fully functional in regards to twitching motility and MP22 infection (**Figure 4A**). As shown in **Figure 4B**, however, the G96S mutant was resistant to PP7 infection, whereas the A95T mutant was PP7-susceptible. This is most likely due to the inability to bind to the PP7 virions (**Figure 4C**). These results led us to the conclusion that the 96th glycine of the PAO1 pilin might be necessary for PP7-TFP interaction.

## Discussion

This is the first study to define a molecular determinant in the TFP pilin that primarily affects the host-spectrum of the TFP-specific leviphage, PP7. The comprehensive investigation on the host-spectrum, the pilin groups of 25 twitching-proficient *P. aeruginosa* strains, and the heterologous pilin expression revealed that PP7 might be able to use a set of the group II (i.e., PP7-susceptible or group IIa) pilins for its infection. We have elucidated a pilin determinant involved in the PP7 susceptibility of group IIa pilin based on the comparison of the amino acid sequences of group IIa and group IIb (i.e., PP7-resistant) pilins: All the group IIa pilins were clustered at a clade with several branches of diversity, whereas the group IIb pilins were clustered together except for the PAK pilin.

It is certain, however, that the PAK pilin renders the PP7 resistance when it is functionally expressed in the PAO1 *pilA* mutant as assessed by the phenotypic characterization of twitching motility and phage sensitivity. This is interesting in that both PAO1 and PAK are relatively well-characterized strains among the *P. aeruginosa* isolates, and more importantly, in that both PAO1 and PAK pilins were relatively similar in amino acid sequences. The variations of these sequences were striking only at the three variable regions of TFP pilins. Based on these results, we propose that pilin sequence variation of *P. aeruginosa* strains is the primary determinant for PP7 susceptibility, which also indicates that the structural moieties and/or amino acid residues of the phage particle need to be engineered in a way to modulate or broaden the host range of PP7 as an antibacterial modality in *P. aeruginosa*. This future venue will have to be accompanied by further identification of the structural determinants in the TFP, which is a helical polymer consisted of the pilin protein, as well as in the phage particles, which are consisted of 180 molecules of the single capsid protein.

In the first full-fledged attempt to identify the determinants in TFP that makes difference between the PAO1 and the PAK pilins, five chimeric pilins between both pilins had been created (Supplementary Figure S1). Unfortunately, these chimeras were not functional, in that they could not rescue the twitching defect of the PAO1 *pilA* mutant. Instead, we have tried to identify mutants for altered susceptibility to PP7 with functional twitching. This is based on a random chemical mutagenesis using EMS to isolate a PAO1 pilin mutant which remains twitching-proficient but PP7-resistant. One such mutant we isolated contained two codon changes at the β1-β2 loop region (A95T/G96S mutation), suggesting that the β1-β2 loop in the V2 region might be crucial in PP7-susceptibility. Since the A95T mutant displayed the same phenotype as the wild type pilin and the G96S mutant represented the phenotype of the original mutant with A95T/G96S mutation, indicating that G96 is one of the key residues in the interaction with PP7, but not for the TFP function. It is quite probable that the complicated structural variations at the β1-β2 loop region may be important in PP7-TFP interaction instead of simple sequence variation. Comparative structural characterization of the group IIa and group IIb pilins will provide insight into the structure-function relationship of TFP, especially at the β1-β2 loop region. Their implications for general and specific functions of both pilin groups will improve our knowledge about their physiological roles in bacterial pathogenesis and therapeutic targets as one of the major virulence factors. The growing knowledge based on the structure-function relationships of TFP will enable us to do the rational design in order to develop a platform to engineer bacterial pilins, which can be exploited in academic as well as commercially applicable uses.

Furthermore, TFPs are relatively conserved among many Gram-negative bacteria, and are interchangeable between different genera. For example, the pilin protein from *Dichelobacter nodosus*, the causative agent of ovine foot rot, was functionally expressed as TFP in *P. aeruginosa* (Elleman et al., 1986). The heterologously expressed and assembled *D. nodosus* TFP engenders immunity to ovine foot rot and thus the recombinant *D. nodosus* TFP remains in use today as a vaccine toward the disease (Elleman and Stewart, 1988). This same methodology was also exploited with success in a number of studies with the pilins from *Moraxella bovis* (Beard et al., 1990), *Neisseria gonorrhoeae* (Hoyne et al., 1992), *E. coli* (Sauvonnet et al., 2000), and *Pseudomonas syringae* (Roine et al., 1998) that were functionally expressed in *P. aeruginosa*. This functional interchangeability in combination with the aforementioned structure-function relationship of *P. aeruginosa* pilin regarding its function as the phage receptor will help broaden the host spectra of PP7.

This information will be also useful in the design of new intelligent synthetic RNA phages based on PP7, which will be geared to develop a new therapeutic bioantibacterial platform with improved antibacterial efficacy or modulated host range. A recent study also hints to another layer of antibacterial activity of an RNA phage, in that it may lead to virulence attenuation due to bacterial mutations in TFP or lipopolysaccharide biogenesis (Pourcel et al., 2017). Based on these, we hope to further the pursuit of new eco-friendly and safe antibacterial modalities which are alternative and supplementary to the current antibiotic regimens in the era of antibiotic resistance.

## Author Contributions

Y-HC conceived and designed the research. ESK and H-WB designed and performed the experiments, and collected and analyzed the experimental data. Y-HC and ESK wrote the manuscript. All authors reviewed the manuscript.

## Conflict of Interest Statement

The authors declare that the research was conducted in the absence of any commercial or financial relationships that could be construed as a potential conflict of interest.
