# Supplementary material for: A Pilin Region Affecting Host Range of the Pseudomonas aeruginosa RNA Phage, PP7
Source: Front Microbiol. 2018 Feb 16;9:247. doi: 10.3389/fmicb.2018.00247 (PMC5820433; doi:10.3389/fmicb.2018.00247)
Supplement: Supplementary file 1 [file Data_Sheet_1.DOCX]

**Supplementary Information**

A pilin region affecting host range of the *Pseudomonas aeruginosa* RNA phage, PP7

Eun Sook Kim,^†^ Hee-Won Bae,^†^ and You-Hee Cho^*^

*Department of Pharmacy, College of Pharmacy and Institute of Pharmaceutical Sciences, CHA University, Gyeonggi-do 13488, Korea*

^*^ Corresponding author.

Phone: 82-31-881-7165.

Electronic mail: youhee@cha.ac.kr

^†^E.S. Kim and H.-W. Bae contributed equally to this work.

Running Title: TFP pilin region and phage susceptibility

Keywords: *Pseudomonas aeruginosa*, RNA phage, PP7, type IV pilus (TFP), pilin, twitching

**
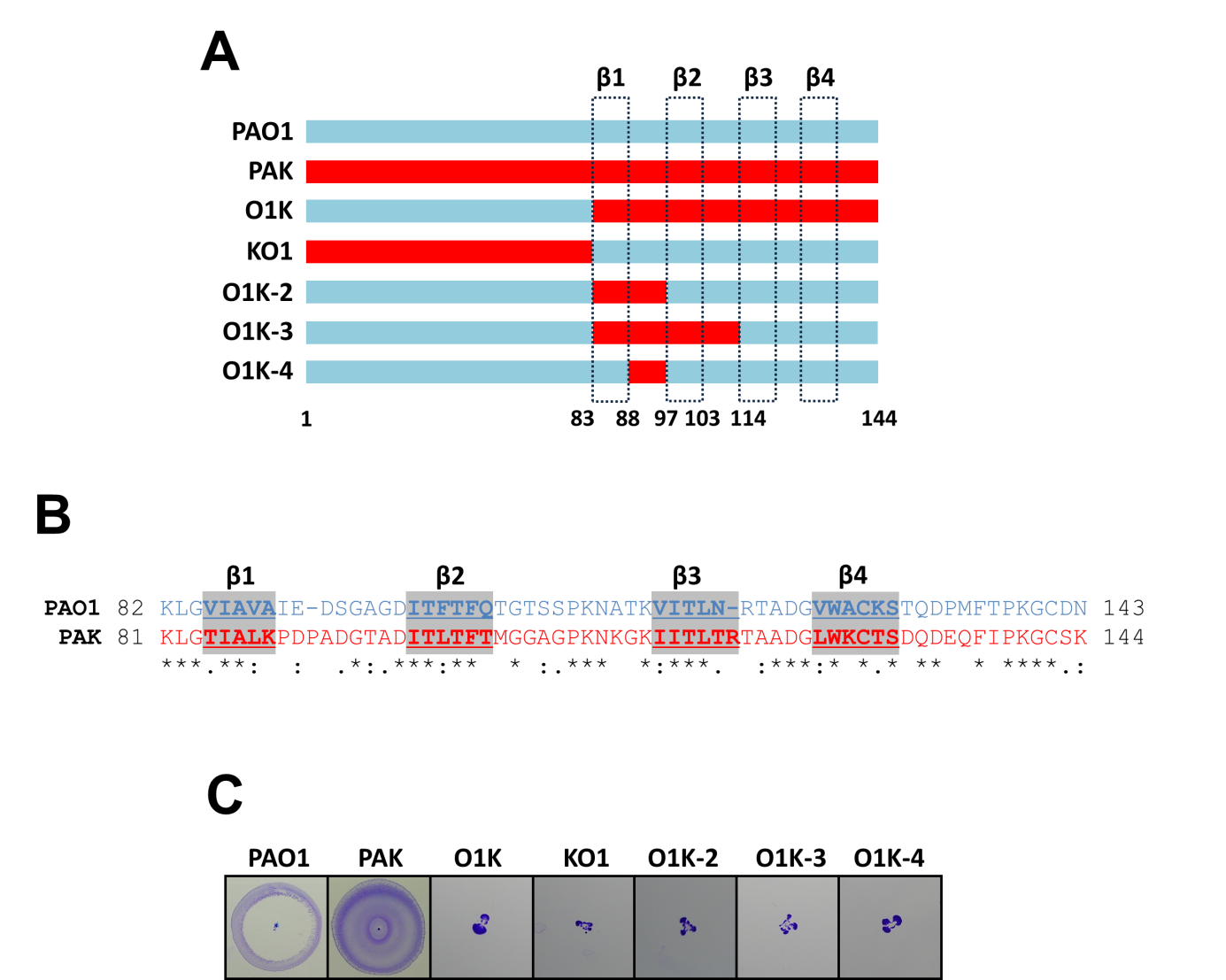
**

**FIGURE S1. Construction of chimeric pilins.**

(A) PAO1-PAK chimeric pilins were generated by SOEing PCR. The five chimeric pilins (O1K, KO1, O1K-2, O1K-3, and O1K-4) were generated, considering that the N-terminal half of the group II contains highly conserved region, whereas the C-terminal half contains the three variable regions (β1-β2, β2-β3, and β3-β4). The internal numbers (83~114) indicate the amino acid number of the PAK pilin at the junction regions of the chimeric pilins.

(B) The amino acid sequences in the junction regions of the chimeric pilins in A. The β1-β4 sheets are underlined in box and the identical residues between PAO1 and PAK pilins are designated by asterisk (*).

(C) The twitching motility of PAO1, PAK and five chimeric pilins. Photographs were taken after 24 h using twitching assay plates.

**Table S1. Bacterial strains used in this study**

| **Strain** | **Relevant characteristics or purpose^a^** | **Reference or source** |
| --- | --- | --- |
| *E. coli*  DH5α  S17-1  *P. aeruginosa*  PAO1  PA14  PAK  57RP  PMM1-53  PAO1 *pilA*  PA14 *pilA*  PAK *pilA* | multi-purpose cloning  conjugal transfer of mobilizable plasmid; Tp^R^; Sm^R^  Wild-type laboratory strain  Wild-type laboratory strain; Rif^R^  Wild-type laboratory strain  Environmental isolate  Clinical isolates from various Asian countries  PAO1 with in-frame deletion of *pilA*  PA14 with in-frame deletion of *pilA*; Rif^R^  PAK with in-frame deletion of *pilA* | Lab collection  Lab collection  Lab collection  Lab collection  Lab collection  Lab collection  Lab collection  This study  1  This study |

**^a^** Tp^R^, trimethoprim-resistant; Sm^R^, stremptomycin-resistant; Rif^R^, rifampin-resistant

**Table S2. Primers used in this study**

| **Primer** | **Sequence^a^** |
| --- | --- |
| pilA-N1  pilA-C1  pilA-C2  pilA-UC(PAO1)  pilA-DN(PAO1)  pilA-UC(PAK)  pilA-DN(PAK)  pilA-UC(PA14)  pilA-DN(PA14)  I90P-UC  I90P-DN  A95T-UC  A95T-DN  G96S-UC  G96S-DN | 5’-ATTACGAATTCGCTGCTCTACCG-3’  5’-AGCGAAAGCTTGTTGCGCTGSGC-3’  5’-CTGGATCGGAAGCTTGGCCACTTC-3’  5’-CATGAATCTCTCCGTTGATTATG-3’  5’-CATAATCAACGGAGAGATTCATGGCCAACAAGTTGGGTGTAATTGC-3’  5’-CATGAATATCTCCATTGATATGT-3’  5’-ACATATCAATGGAGATATTCATGGCTAACAAACTGGGTACTATCGC-3’  5’-ACGAATGGTGTAATCCTG-3’  5’-CAGGATTACACCATTCGTGAAGAGTTGATGAGGTTT-3’  5’-GGGTGCTACAGCAATTAC-3’  5’-ATTGCTGTAGCACCCGAAGAT-3’  5’-GTGGTACT GGTGATATTA-3’  5’-CACCAGTACCACTATCTT-3’  5’-ACTCGCACCACTATCTTC-3’  5’-GGTGCGAGTGATATTACC-3’ |

**^a^** underline denotes the engineered restriction enzyme sites

Reference

1. Heo, Y.-J., Chung, I.-Y., Choi, K. B., Lau, G. W. & Cho, Y.-H. Genome sequence comparison and superinfection between two related *Pseudomonas aeruginosa* phages, D3112 and MP22. Microbiology 153, 2885-2895 (2007).
